# CAR T cells remain during long-term cancer remission

**DOI:** 10.1038/s43856-022-00092-w

**Published:** 2022-03-11

**Authors:** Katharine Barnes

**Affiliations:** Communications Medicine, https://www.nature.com/commsmed

## Abstract

A number of chimeric antigen receptor (CAR) T cell-based therapies are now approved by the FDA for the treatment of cancer. A study published in *Nature* found that CAR T cells are still present in two patients who remain cancer-free over a decade after they received CAR T cell therapy as part of a clinical trial.

**Figure:**
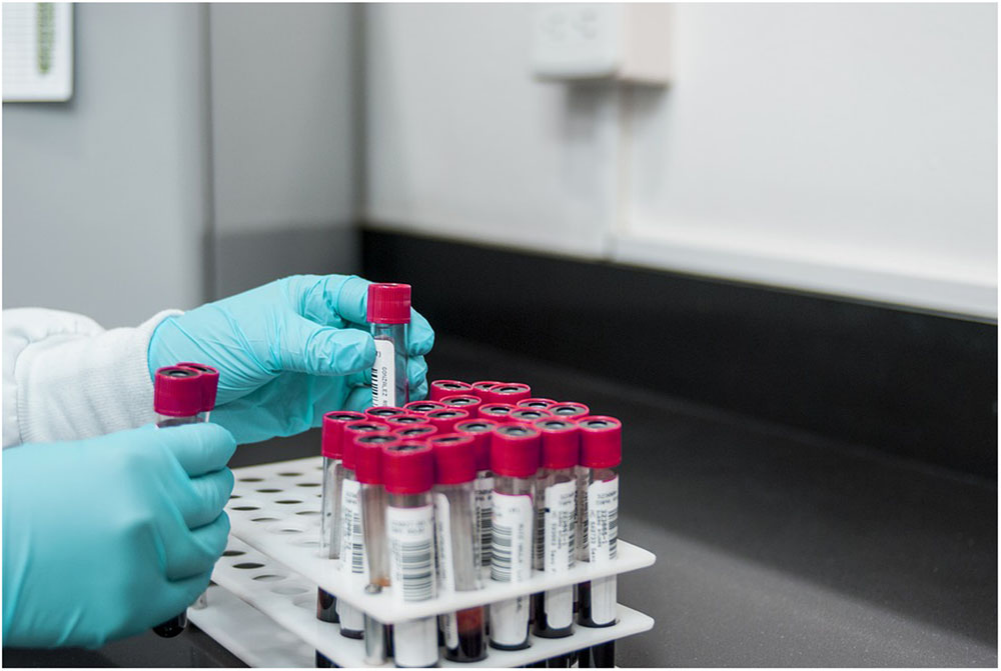
Pixabay

Chronic lymphocytic leukaemia (CLL) is a cancer that arises in B cells, the cells within the immune system that produce antibodies, and is one of the most common types of leukaemia in adults. Chimeric antigen receptor (CAR) T cell therapy, in which a patient’s T cells are isolated and genetically engineered to attack the patient’s tumour cells, has been tested as a treatment in patients with CLL. A subset of CLL patients treated with CAR T cells targeting the B cell antigen CD19 (CTL019 cells) as part of a phase I trial in 2010 exhibited complete and durable responses, with some still in remission over 10 years later.

To further understand the biology of such sustained responses to CAR T cell therapy, Melenhorst et al. studied two of the patients who displayed complete responses to CTL019 cells in 2010 and remain in remission^1^. The authors investigated whether CTL019 cells remained detectable in the patients. Peak numbers of CTL019 cells occurred in the patients 3 and 31 days after infusion. However, 10 and 9 years post-infusion, CTL019 cells remained, representing 0.8 and 0.1% of all T cells analysed from the two patients.

By sequencing the T cell receptor on the CAR T cells, the authors showed that the specific populations of CTL019 cells present in the patients changed initially but then stabilised at different time points in the two patients. Two major subtypes of T cells are CD4+ (helper) T cells and CD8+ (cytotoxic) T cells, which express either the CD4 or CD8 glycoproteins. Initially, the CTL019 cells lacked either glycoprotein or were CD8+; later, a small number of CD4+ cell populations became dominant. Further analysis of these CD4+ T cells suggested that they remained functionally active and, unusually, had some of the characteristics of cytotoxic T cells.

In summary, these results show that there have been two major phases of response in the patients, with an initial response phase followed by a long-term remission phase. The sustained remission seen in these two patients could be a consequence of the cytotoxic activity of persistent CTL019 cells. Studies such as this one further our mechanistic understanding of how these treatments work long-term and elicit the remarkable clinical outcomes seen in some patients.
